# Cloud–Edge Collaborative Personalized Deployment of Knowledge Bases in Semantic Communications

**DOI:** 10.3390/s26165299

**Published:** 2026-08-21

**Authors:** Kaixiang Yang, Yushen Han, Yikai Xu, Mingkai Chen

**Affiliations:** The Key Laboratory of Broadband Wireless Communication and Sensor Network Technology, Nanjing University of Posts and Telecommunications, Nanjing 210003, China; 1225013624@njupt.edu.cn (Y.H.); 1222014518@njupt.edu.cn (Y.X.); mkchen@njupt.edu.cn (M.C.)

**Keywords:** optimization deployment, SKB, cloud–Edge collaboration, semantic communications

## Abstract

With the rapid evolution of next-generation mobile communications, semantic communication has emerged as an intelligent communication paradigm capable of surpassing the Shannon limit. A fundamental prerequisite for this paradigm is the synchronization of background knowledge between the transmitter and receiver, making the semantic knowledge base (SKB) a critical cornerstone. However, effectively selecting appropriate content from massive cloud-based knowledge repositories for edge deployment remains a significant challenge. This paper conducts systematic research to address the key issues in the flow deployment of SKBs at the edge, including insufficient adaptation to personalized preferences, inadequate timeliness management, and the complexity of multi-objective optimization. First, a comprehensive system model is constructed, integrating user preferences, knowledge relevance, transceiver matching degree, and the Age of Information (AOI). Second, the Generative Adversarial Network (GAN)-assisted Preference-based Reinforcement Learning (GaPbRL) algorithm is proposed. The experimental results demonstrate that this method outperforms traditional schemes in terms of knowledge-base hit rate, transceiver matching degree, and algorithm convergence speed, while significantly reducing the overhead of manual fine-tuning. This study provides a robust framework for the personalized and efficient cloud–edge collaborative deployment of SKBs.

## 1. Introduction

With the vigorous advancement of next-generation mobile communications, traditional communication systems based on Shannon’s information theory are increasingly encountering inherent performance bottlenecks. Semantic communication [1,2], as an innovative intelligent communication paradigm, transcends the constraints of bit-level transmission by prioritizing the delivery of the core information semantics over redundant raw data. By integrating communication with intelligence, this approach reshapes critical technical processes, including information extraction, semantic representation, encoding, decoding, and content reconstruction [3]. As a promising technology poised to break the Shannon limit, semantic communication has evolved into a key research direction in future network domains.

Currently, SKBs [4] have achieved significant progress in three technical dimensions, knowledge extraction, dynamic management, and performance optimization, thus overcoming the bottlenecks of traditional applications. In [5], the SKB serves as the fundamental cornerstone of efficient semantic communication, since effective semantic interaction requires that the sender and the receiver have consistent background knowledge. It encompasses structured entities, relational associations, and task-specific domain knowledge, providing critical support for semantic comprehension, logical reasoning, and accurate decoding throughout the communication process. Whether resolving semantic ambiguities or supplementing contextual semantic details, the rationality of its deployment and the effectiveness of its application directly determine the overall performance of the semantic communication system, including communication accuracy, timeliness, and quality of experience (QoE). In [6], a joint construction scheme that utilizes SKBs and large language models (LLMs) has demonstrated the ability to defend effectively against poisoning attacks.

Moreover, the performance of an SKB [7] is governed primarily by two core dimensions: completeness and preference responsiveness. Completeness refers to the extent to which the knowledge base covers semantic information relevant to the task, including the richness of entity sets, the accuracy of relational descriptions, and the comprehensiveness of attribute information. Incomplete knowledge bases often induce semantic ambiguity or information loss, thereby undermining reliability. In contrast, preference responsiveness reflects the knowledge base’s capacity to adapt to individual user demands and dynamic application scenarios. Different users exhibit divergent backgrounds and usage preferences that may evolve dynamically over time. A knowledge base incapable of accommodating these personalized and time-varying preferences will inevitably lead to mismatches between communication content and user needs [8]. Balancing completeness with flexible preference responsiveness constitutes a key technical challenge in current research. In [9], LLMs are leveraged to construct a novel communication framework. The rise of LLMs provides core technical support for the construction and optimization of SKBs. As artificial intelligence models with massive parameters and powerful semantic understanding capabilities, LLMs can automatically extract structured entities, relationships, and task-related knowledge from large-scale unstructured data, efficiently complementing semantic associations while significantly reducing manual construction costs. Meanwhile, LLMs possess excellent contextual reasoning and preference mining capabilities, enabling them to dynamically identify personalized needs from interaction data and provide accurate references to optimize responsiveness [10]. Their generative capabilities also assist in resolving semantic ambiguities and supplementing context-dependent details—for instance, generating multi-dimensional interpretations for ambiguous expressions to align the background knowledge of communication parties. Furthermore, LLMs can be deeply integrated with dynamic deployment mechanisms: by analyzing the timeliness of knowledge elements and shifting user needs in real time, they offer intelligent decision support for AoI-driven update and replacement strategies, thereby further enhancing the freshness and adaptability of edge-side SKBs.

To address the two main issues in the deployment of SKBs [11]—insufficient personalized preference matching and a lack of knowledge timeliness management—the AoI is integrated into the proposed dynamic deployment framework. As a key metric for quantifying the freshness of the information, AoI is defined as the time elapsed since the generation of the knowledge-base data. Traditional static deployment methods typically optimize only for cache hit rates, ignoring heterogeneous user preferences, causal correlations between knowledge entities, and the degree of matching between the sender and receiver, which is prone to issues such as outdated knowledge and semantic ambiguity. In contrast, the AoI-incorporated dynamic deployment method adjusts strategies for retaining, deleting, and replacing edge-side knowledge by monitoring the timeliness of elements in real time. Adapting to personalized needs ensures high knowledge freshness, effectively mitigates semantic deviations caused by outdated information, and ultimately improves the comprehensive performance of the system.

SKBs exhibit prominent multi-layered and multi-preference characteristics in practical applications [12]. The multi-layered nature is reflected in the classification of knowledge into shared public knowledge and personalized private knowledge. The multi-preference characteristic manifests itself as the diverse and evolving interests of different users [13]. These inherent characteristics give rise to an intrinsic trade-off between SKB scale and operational efficiency: expanding the scale improves completeness but imposes excessive storage burdens on edge devices and reduces query and update efficiency. In contrast, reducing the scale to optimize efficiency may result in insufficient coverage of key knowledge [14]. Balancing this trade-off to achieve efficient operation under constrained edge resources has become an urgent technical issue.

- The SKB edge deployment must simultaneously satisfy four core requirements with inherent trade-offs: (1) adaptation of personalized preferences; (2) semantic consistency; (3) information timeliness; (4) constraints on edge resources. Traditional edge caching methods mostly focus on a single objective and lack systematic design for multi-objective optimization, making it difficult to achieve a dynamic balance among these factors.
- The reward engineering needs to integrate multi-dimensional indicators, e.g., hit rate, matching degree, and AoI, where manually quantifying weights is difficult and prone to reward hacking. However, implementing reinforcement learning from human feedback (RLHF) requires a large volume of expert annotations for trajectory preferences, which involves high costs, low efficiency, and limited generalization. This makes it difficult to adapt to universal semantic communication scenarios.

Compared with standard deep reinforcement learning and vanilla PbRL methods, the distinct technical contributions of this work are summarized as follows:

1. We introduce a GAN-driven preference expansion mechanism that encapsulates human-expert evaluation logic into a stable mathematical distribution. By substituting active human labeling with simulated adversarial evaluations, our framework achieves data-driven completeness and breaks the scalability bottleneck of traditional preference learning in dynamic communication networks.
2. Unlike conventional edge caching that focuses entirely on data-level hit rates, our framework jointly optimizes semantic comprehension alignment (transceiver matching degree) and information staleness (AoI) under limited edge storage capacities.

The structure of this paper is as follows. First, in Section 2, we introduce the system model for collaborative deployment of SKBs on the cloud edge, including the representation form and the definition of key metrics. Next, we formulate the optimization problem, clarifying the objective function and constraints. Subsequently, in Section 3, we elaborate on the detailed design of the GaPbRL method, covering MDP modeling, GAN-assisted preference expansion, and policy learning. In Section 4, we present simulation experiments that verify the superiority of the proposed method. Finally, in Section 5, we summarize the core research findings.

## 2. System Model

### 2.1. Scenario

A significant distinction between semantic communication systems and conventional communication systems is that semantic communication systems require an SKB that conforms to the personalized preferences of communication users. This section first introduces the performance gains brought about by knowledge bases in semantic communication systems. At the transmitting end, the semantic information *S* is encoded in a signal *X*. After transmission through the channel, the received signal *Y* is obtained, from which the receiver decodes the estimated semantic information $\hat{S}$. Defining the decoding precision as $F(S,\hat{S})$, mutual information as I(S;Y), and the knowledge base as *K*, from the perspective of information theory the literature [14,15] demonstrates that

(1) $$\begin{array}{c} F\left(S,\hat{S}|K\right)\geq F\left(S,\hat{S}\right), \end{array}$$

(2) $$\begin{array}{c} I(S;Y|K)\leq I(S;Y). \end{array}$$

These expressions imply that an SKB-assisted system improves the accuracy of encoding and decoding model training while reducing transmission overhead [16]. To theoretically formalize the performance gain of limiting the information age, let $F(S,\hat{S}|K)$ denote the decoding precision conditioned on the deployed knowledge base *K*. Because semantic information fields drift dynamically over time, the real-world utility and accuracy of edge-cached knowledge decline as a function of the elapsed time since its generation in the cloud. Let $\Delta f\left(t\right)=f_{n}\left(t+1\right)-f_{n}\left(t\right)$ be the variation in knowledge age. The instantaneous semantic hit rate η(t) degrades according to an exponential decay model, formulated as

(3) $$\Delta \eta \left(t\right)=k_{3}e^{\Delta f(t)}+k_{4},$$

where $k_{3}<0$ is a negative scaling constant determined by the dynamic volatility of the specific knowledge category. This formulation guarantees that constraining the average age to $f_{n}\left(t\right)\leq \lambda$ mathematically minimizes the semantic ambiguity boundary and protects the transceiver matching degree C(t) from sudden drop-offs. However, maintaining a singular centralized SKB is prohibitively expensive, and directly deploying large-scale centralized knowledge bases to the edge is similarly impractical. Taking into account the graph-structured characteristics of semantic data [17], this paper focuses on introducing how the SKB is collaboratively deployed at the edge and in the cloud [18].

**Figure 1:**
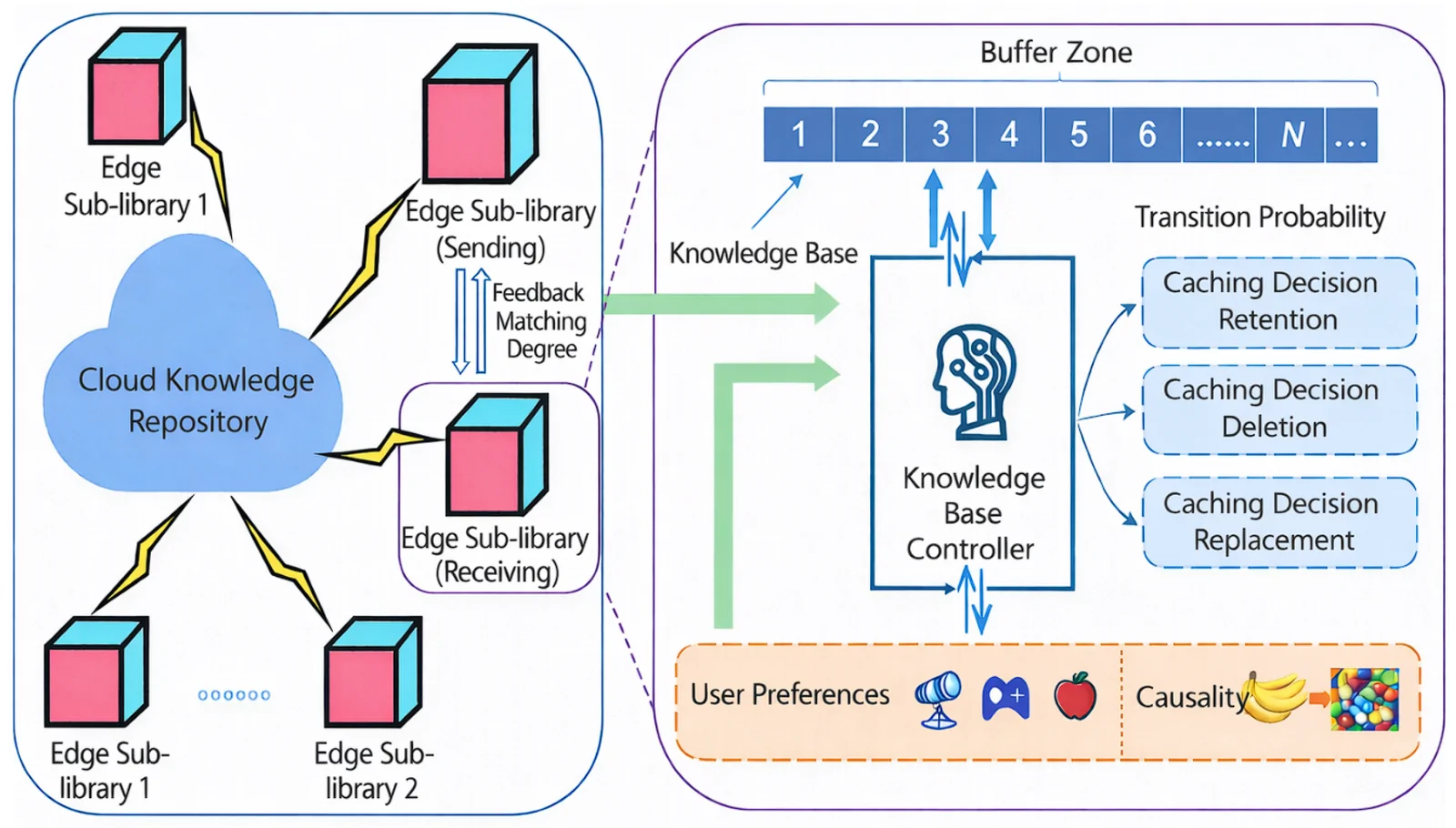
SKBs in cloud–edge collaboration for semantic communications.

Figure 1 illustrates the cloud–edge collaborative architecture for semantic communications. The specific knowledge sub-bases cached on the edge server are collectively referred to as edge sub-bases, whereas the cloud-side repository is defined as the cloud knowledge master base [19]. Due to the limited storage capacity of edge servers, the content of edge sub-bases is selectively extracted from the master base and organized by categories. Semantic codecs can be trained specifically according to distinct semantic knowledge categories [20]. The edge sub-base is assumed to support up to *N* distinct categories [21]. A buffer zone is structurally established at the edge server–node interface. The primary function of the buffer zone is to serve as an asynchronous staging cache that temporarily accommodates incoming knowledge chunks streamed dynamically from the cloud master repository during cache misses or updates. By acting as a traffic smoothing layer, it buffers variations in transmission throughput and prevents cloud–edge synchronization latencies from disrupting the local, real-time semantic codec pipelines executing on edge devices.

During initialization, the edge server bootstraps its local semantic cache by selectively loading knowledge sub-bases that simultaneously align with historical user preferences and the factual relevance of anticipated data requests, subject to the available storage budget. Whenever a mobile device issues a semantic-based understanding request [22], in [23] the server queries its resident knowledge base. In the event of a cache failure, the server streams the required segment from the cloud and updates the local repository atomically to ensure seamless downstream inference.

The system continuously monitors observable metrics, including user preferences, causal relationships, sender–receiver SKB affinity, and instantaneous cache occupancy. These metrics are utilized to recalibrate the cache at the start of every time slot. Oriented by the matching affinity, the remaining capacity, and the AoI of each data chunk, the edge controller decides which semantic partitions to retain, evict, or reuse, thus formulating the SKB caching problem as a constrained Markov decision process (CMDP) [24].

In this paper, we assume that each user device corresponds to an edge server. The knowledge base on the cloud end is defined as $G_{\mathrm{total}}=\left\{V\left(G_{\mathrm{total}}\right),E\left(G_{\mathrm{total}}\right),L\right\}$, where the knowledge base stored on the sending edge server is represented by $G_{s}=\left\{V\left(G_{s}\right),E\left(G_{s}\right),L_{s}\right\}$; and the knowledge base stored on the receiving edge server is $G_{j}=\left\{V\left(G_{j}\right),E\left(G_{j}\right),L_{j}\right\}$. The relationship between the subsets *g* and the total set *G* is $g_{1}\cup g_{2}\cup \ldots \cup g_{N}=G$. V(G) represents the set of entities, including source entities and target entities, referring to entities or concepts of the real world, and E(G) represents the relationships between different entities. L(G) represents the label of the knowledge base, which is automatically generated when the knowledge base is extracted [25]. The label consists of multiple components, primarily including the category and the data volume of the knowledge base. For a given category, the extraction degree of the master knowledge base at the cloud server is maintained at 100%. The timeliness requirement of the knowledge base of this category, quantified as the value of the AoI weight *w*, and a current timestamp are generated when transmitted to the edge server to record the AoI.

In this paper, we define user preferences as related to the frequency of the user’s requests for corresponding categories of knowledge bases. Each user’s preference can be represented by a preference label $P=[p_{1},p_{2},\ldots ,p_{N}]$, where the value of each element represents the user’s degree of preference for the content feature label, as the probability of being ranked at the top. It is assumed that the probability of a user’s preference for a certain category of content follows a Zipf distribution with parameter ϕ [26], and its calculation can be expressed as

(4) $$\begin{array}{c} p_{n}\left(t\right)=\frac{{\left[y_{n}\left(t\right)\right]}^{-\phi }}{\sum_{n\in N}{\left[y_{n}\left(t\right)\right]}^{-\phi }},\;\phi \geq 0, \end{array}$$

where *n* represents the descending rank of the access frequency of the knowledge base of the *n*-th category at time *t*, and ϕ is a real constant used to control the degree of aggregation of the interest of the knowledge base [27]. A larger ϕ indicates that the knowledge base with a higher rank has a higher request frequency.

We define a relevance label to determine the causal relationship of the content browsed by the user. The relevance label is expressed as $q=(q_{1},q_{2},\ldots ,q_{h})$. The causal relevance between two categories of knowledge bases is expressed using cosine similarity. We assume $I=(I_{1},I_{2},\ldots ,I_{N})$ represents the content of various knowledge sub-bases stored on the edge server, where *I* is an *N*-dimensional vector, with each dimension representing a knowledge-base category. The relevance between category *m* and category *n* is expressed as

(5) $$\begin{array}{c} q_{t}\left(m,n\right)=\frac{I_{m}\left(t\right)\cdot I_{n}\left(t\right)}{∥I_{m}\left(t\right)∥∥I_{n}\left(t\right)∥}. \end{array}$$

Consequently, the knowledge-base hit rate at time *t*, η(t), is defined as

(6) $$\begin{array}{c} \eta \left(t\right)=k_{1}\sum_{n}p_{n}\left(t\right)+k_{2}\sum_{m}\sum_{n}q_{t}\left(m,n\right). \end{array}$$

Furthermore, the degree of matching of the knowledge base C(t) is defined as the sum of the ratios of the degrees of extraction *M* of the corresponding categories of knowledge bases [28] extracted by the receiver edge server from the cloud and is expressed as

(7) $$\begin{array}{c} C\left(t\right)=\sum_{n=1}^{N}\frac{M_{n}^{r}\left(t\right)}{M_{n}^{s}\left(t\right)}. \end{array}$$

Considering that the receiver optimizes extraction based on private parameters, the degree of extraction $M_{n}^{r}\left(t\right)$ is typically smaller than the degree of the sender $M_{n}^{s}\left(t\right)$. To quantify the efficiency of an SKB over time, we employ the AoI, f(t):

(8) $$\begin{array}{c} f\left(t\right)=t-t_{0}, \end{array}$$

where *t* is the current time and $t_{0}$ is the generation timestamp in the cloud. To ensure data freshness, an AoI threshold λ is defined [29]. Sub-bases exceeding this threshold are updated, triggering operational adjustments such as modified extraction degrees. Importantly, for communication services, AoI requirements may deviate from conventional definitions; while edge interfaces require dynamic adjustment, general-purpose knowledge may not necessitate extreme freshness. However, personalized sub-bases tailored to user preferences are highly dynamic and require a low AoI to maintain system efficiency [30].

In this paper, the maximum value of the AoI knowledge base is assumed to be $f_{n}\left(t\right)\in \left\{1,2,\ldots ,\lambda \right\}$. When $f_{n}\left(t\right)$ exceeds the threshold, it indicates that the knowledge base stored in the edge server’s *n*-th category is inefficient, which may result in generating ambiguous meanings. The equation can be expressed as follows:

(9) $$\begin{array}{c} f_{n}\left(t+1\right)=\left\{\begin{array}{cc} \;\;1, & a_{n}\left(t\right)=A_{t},\\ \min\left\{\lambda ,f_{n}\left(t\right)+1\right\}, & \mathrm{otherwise}. \end{array}\right. \end{array}$$

where $a_{n}\left(t\right)$ represents the action of the intelligent body in the knowledge base of the *n*-th category at time *t*, and $a_{n}\left(t\right)=A_{t}$ indicates that the intelligent body is performing action $A_{t}$ in the knowledge base of the *n*-th category at time *t*. Under the operation of strategy π, starting from the initial state s(0), the long-term average AoI of the knowledge base of the *n*-th category is expressed as

(10) $$\begin{array}{c} \bar{f_{n}\left(t\right)}≜\underset{d\to \infty }{\mathrm{lim sup}}\frac{1}{d+1}\sum_{t=0}^{d}\left[f_{n}\left(t\right)|s\left(0\right)\right]. \end{array}$$

Therefore, the upper limit for the AoI is represented by $\bar{f_{n}\left(t\right)}\leq \sigma$ and $\sum_{n}w_{n}\bar{f_{n}\left(t\right)}\leq \psi$, where σ represents the average value of the AoI of each knowledge base. $w_{n}$ represents the weight of the *n*-th knowledge base, and $w_{n}\geq 0$, with $\sum_{n}w_{n}=1$. The weight can be assigned by the edge server based on the importance of the different categories of the knowledge base [31]. When focusing on the efficiency of knowledge bases, a lower AoI weight is assigned to commonly used knowledge bases, while higher AoI weights are assigned to more dynamic, personalized knowledge bases that require more attention to be given to their efficiency.

### 2.2. Problem Formulation

Taking into account the constraints of cache capacity and AoI, the objective function of this paper is given as follows:

(11) $$\begin{array}{cc} \max\;\; & \lim_{T\to \infty }\frac{1}{T}\sum_{t=0}^{T-1}\left[\alpha C(t)+\beta \eta (t)\right]\\ s.t.\;\; & C1:\;\sum_{n}\left[x_{n}\left(t\right)D_{n}\left(t\right)\right]\leq S,\\ \; & C2:\;\sum_{n}x_{n}\left(t\right)\leq N,\\ \; & C3:\;x_{n}\left(t\right)\in \left\{0,1\right\},\;\forall n\in \left\{1,2,\ldots ,N\right\},\\ \; & C4:\;0\leq M_{n}^{r}\left(t\right)\leq M_{n}^{s}\left(t\right)\leq 1,\\ \; & C5:\;\bar{f_{n}\left(t\right)}\leq \sigma ,\\ \; & C6:\;\sum_{n}w_{n}\bar{f_{n}\left(t\right)}\leq \psi ,\\ \; & C7:\;\sum_{n}w_{n}=1,\;\forall w_{n}\geq 0,\\ \; & C8:\;0\leq f_{n}\left(t\right)\leq \lambda ,\;\forall f_{n}\left(t\right)\in Z,\\ \; & C9:\;\alpha +\beta =1,\;\alpha >0,\;\beta >0. \end{array}$$

$x_{n}\left(t\right)$ is a control variable, where if $x_{n}\left(t\right)=1$ it indicates that the corresponding knowledge base is cached, and if not, it is not cached. C1 indicates that the knowledge-base data stored on the edge server do not exceed *S*. C2 indicates that the knowledge-base category stored on the edge server does not exceed *N*. C3 is used to judge whether the knowledge base is cached. C4 limits the knowledge-base extraction process. C5 restricts the AoI of each knowledge base, and the average AoI in the knowledge-base extraction process cannot exceed σ. If the AoI is too large, it will lead to inefficient network usage. C6 represents the weight limit of each knowledge base. C7 is the weight limit for each knowledge base and C8 limits the rights of each knowledge base. C9 is the constraint of the objective function.

The deterministic constraints C1 to C9 mapped from the physical edge architecture are managed via a two-layer safety mechanism within the DRL agent. Hard physical storage limits (C1, C2) are enforced by an action-validation filter: if the proposed action $a_{t}$ causes $\sum_{n}x_{n}\left(t\right)D_{n}\left(t\right)>S,$ the environment rejects the transition, inflicts a structural negative step penalty, and rolls back the agent’s state to re-sample valid actions. Time-freshness boundaries (C5, C6) are explicitly integrated into the CMDP state transition rules, where sub-bases exceeding the threshold λ are dynamically flagged for high-priority eviction.

The objective function reflects that the AoI in the knowledge-base cache problem [32] is constrained by the decision-making process. The smaller the AoI, the better the efficiency of the knowledge base, which can be expressed as the change in the AoI that affects the rate of the knowledge base in the network $\Delta \eta \left(t\right)=k_{3}e^{\Delta f(t)}+k_{4}$. The time-varying behavior of the edge server is modeled by the change in data storage as $\Delta C\left(t\right)=k_{5}\Delta \sum_{n}\left[x_{n}\left(t\right)D_{n}\left(t\right)\right]+k_{6}$, where $D_{1},D_{2},\ldots ,D_{n}$ are the data storage amounts of different knowledge categories. Meanwhile, we prove that the optimization problem in this paper is NP-hard in Appendix A.

## 3. GAN-Assisted Preference Reinforcement Learning for SKB Deployment

### 3.1. Markov Decision Process on SKB Deployment

Given that the optimization problem was proven to be NP-hard in Section 2, deep reinforcement learning (DRL) is used to find the optimal solution. At each time step, the SKB controller determines the currently cached content and evaluates newly acquired content based on the state. We explore an optimal strategy to achieve efficient SKB caching within specific storage constraints [33], aiming to maximize the degree of matching between the receiver and sender knowledge bases. First, the Markov decision process (MDP) and the system state space *S* are defined, where the state at time *t* is denoted as $s_{t}=G_{t}=\left\{g_{1},g_{2},\ldots ,g_{n}\right\}$. The system’s state space encompasses the knowledge content *G*, while the action space *A* is characterized by an initial state distribution μ and a bias rate ρ. The following sections provide a comprehensive explanation of these components [34].

The state space *S* represents the content of the SKB *G*. The state at time *t* is represented as $s_{t}=G_{t}=\left\{g_{1},g_{2},\ldots ,g_{n}\right\}$, which indicates the content of the knowledge base stored on the edge server. From the knowledge content $G_{t}$, we can read the knowledge signature *L*, such as the usage of the knowledge base $\sum_{n}x_{n}\left(t\right)D_{n}\left(t\right)$, the category of knowledge $\sum_{n}x_{n}\left(t\right)$, the moment $t_{0}$, the AoI weight $w_{n}$, and the degree of extraction $M_{n}\left(t\right)$, which is represented as $L=\sum_{n}x_{n}\left(t\right)D_{n}\left(t\right),\sum_{n}x_{n}\left(t\right),t_{0},w_{n},M_{n}\left(t\right)$. $t_{0}$ is the time when the knowledge is transferred to the edge server, used to calculate the AoI of the *n*-th SKB category [35].

The action space $A=\{a_{1},a_{2},\ldots ,a_{n}\}$ corresponds to the set of sub-bases $\left\{g_{1},g_{2},\ldots ,g_{n}\right\}$. Each action *a* is determined by the controller by evaluating the degree of matching between transceivers in conjunction with storage and efficiency constraints. The controller dynamically adjusts the degree of extraction of each SKB. When storage blocks are removed while maintaining the same extraction degree, the system can effectively select newly extracted knowledge for replacement. The controller first observes historical cloud-based requests for different SKB categories. The initial probability distribution model is given by $p_{n}\left(t\right)=\frac{{\left[y_{n}\left(t\right)\right]}^{\varphi }}{\sum_{n\in N}{\left[y_{n}\left(t\right)\right]}^{\varphi }},$ where the categories are ranked according to the user’s preference [36]. The edge device randomly samples content from the cloud to obtain the initial state distribution μ(s). In preference reinforcement learning, a sampled trajectory can be expressed as $\tau =\{s_{0},a_{0},s_{1},a_{1},\ldots ,s_{n-1},a_{n-1},s_{n}\}$ [37]. We define $\tau_{i}≻\tau_{j}$ as meaning that human experts prefer the trajectory $\tau_{i}$ over the trajectory $\tau_{j}$. If $\tau_{i}≻\tau_{j}$, these two trajectories are stored in the SKB preference set (KBPS), ζ, in the form of the triple $\left(\tau_{i},\tau_{j},1\right)$ [38]. $\tau_{i}∼\tau_{j}$ indicates that human experts cannot distinguish the preference between these two trajectories. If $\tau_{i}∼\tau_{j}$, the two trajectories are stored in ζ as the triple $\left(\tau_{i},\tau_{j},0.5\right)$. The preference probability $\rho (\tau_{i}≻\tau_{j})$ is defined as the probability that $\tau_{i}≻\tau_{j}$ given the trajectories $\left(\tau_{i},\tau_{j}\right)$.

### 3.2. Personalized Bias Reinforcement Learning with GAN

This paper incorporates a generative adversarial network (GAN) to assist the preference-based learning method (PbRL) in determining the weighting parameters α and β in the objective function. Subsequently, deep Q-networks (DQNs) are utilized to solve the NP-hard problem. By integrating a normal distribution and expert-guided strategies, the model achieves personalized cloud–edge collaboration [39].

**Figure 2:**
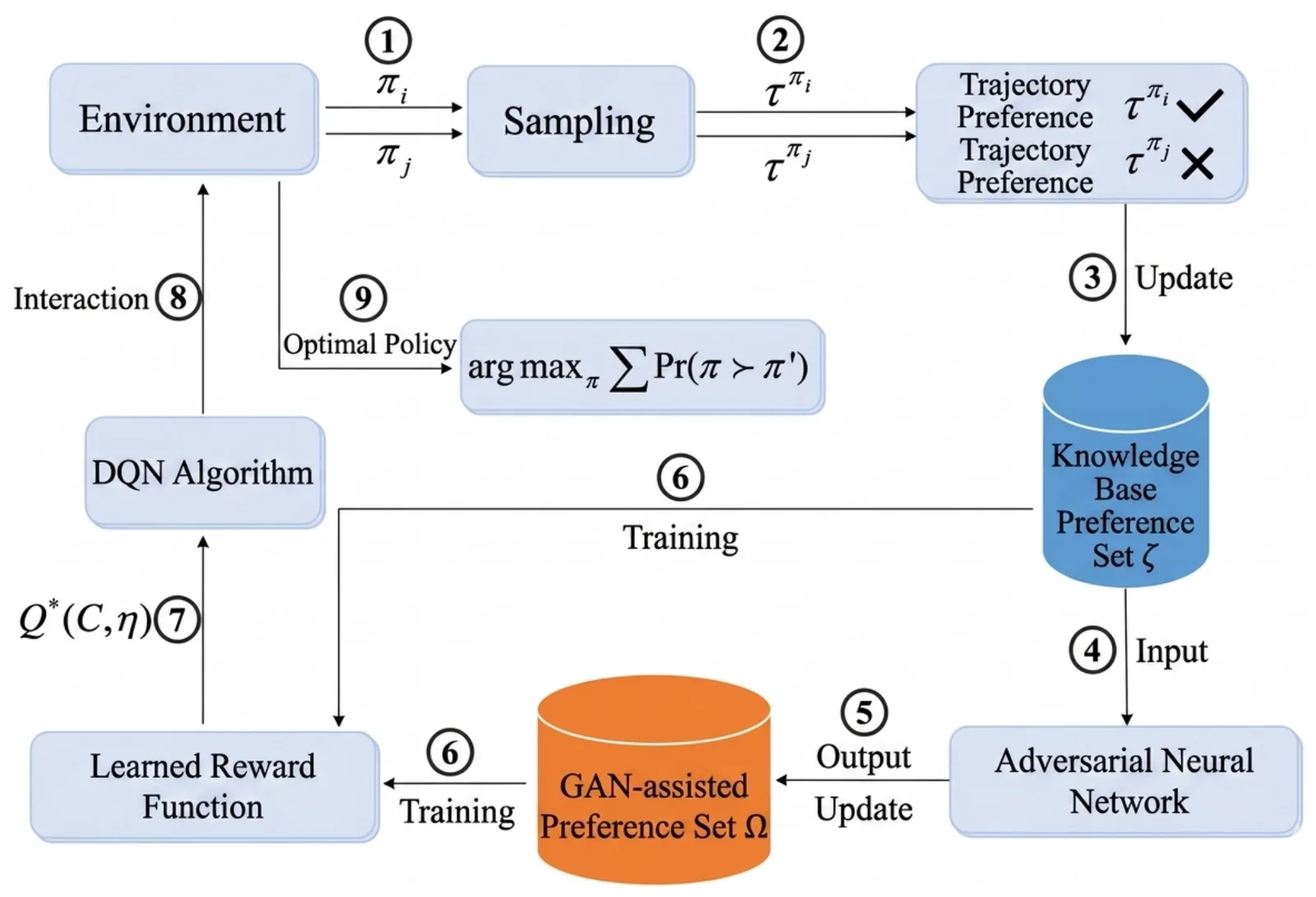
Diagram of the GaPbRL algorithm for SKB cloud–edge collaborative deployment.

The general framework of the algorithm in this paper is shown in Figure 2. The agent selects trajectories from the environment, which are then compared by human experts. To simulate the subjective behavior of human preference, the system processes the set of preferences ζ to train a neural network that emulates human decision-making, producing the set of GAN-enhanced biases Ω. The PbRL network integrates ζ and Ω to train a reward function $Q^{*}\left(C,\eta \right)$, and then a Q function via the DQN algorithm of the Q network, while the DQN algorithm optimizes the policy for the optimal SKB placement strategy. The rationale for adopting a DQN rather than continuous control algorithms like PPO or SAC lies in the inherently discrete nature of the SKB caching action space (retention, deletion, replacement). Furthermore, the weighting coefficients (α and β) were selected via grid search to optimally balance the cache efficiency and matching degree. To validate the robustness of these hyperparameters, a sensitivity analysis was conducted, demonstrating that the system performance remains stable within a ±10% fluctuation of the chosen weights. In Figure 2, the GaPbRL algorithm for the deployment of the collaborative SKB on the cloud edge is shown. The GaPbRL algorithm uses three distinct components of the neural network: the GAN, the PbRL reward generation network, and the DQN [40]. Their interaction follows these procedural rules:

1. Generate the current strategy π for the adversarial network to generate the trajectory $\left(\tau_{n},\tau_{m}\right)$.
2. Update the adversarial network and then map it to the GAN-assisted bias set Ω, where Ω is the set of {1, 2}, representing the trajectory preference.
3. Train the reward model to predict the Q value of human preference.
4. Update the parameters using the DQN algorithm to obtain the new control strategy and maximize the Q value of human preference. This step proceeds with three iterations [41] to stabilize the results generated by the neural networks, i.e., the continuous difference between two returns is less than a threshold. The detailed implementation of the GaPbRL algorithm is shown in Algorithm 1.

**Algorithm 1** GaPbRL Algorithm for Cloud–Edge Collaborative Deployment of semantic knowledge bases**1. Initialization**-Initial Zipf distribution model: $p_{n}\left(t\right)=\frac{{\left[y_{n}\left(t\right)\right]}^{-\varphi }}{\sum_{n\in N}{\left[y_{n}\left(t\right)\right]}^{-\varphi }}\;\;\to \;\;\mu \left(s\right)=\left\{G_{s,0},\;G_{j,0}\right\}$-Satisfy the optimization problems from C1 to C4.**2. Train** α **and** β **in the reward function**
-**Sample**: Select trajectories $\tau^{\pi_{i}},\tau^{\pi_{j}}$ from policies $\pi_{i},\pi_{j}$.-**Query**: Label preferences y∈{0,1} via human expertise.-**Update GAN**: $\theta_{di}\leftarrow \theta_{di}+\nabla_{\theta_{di}}E_{\tau_{i}\in \Omega_{np}}\log\left(1-Y_{di}\left(\tau_{i}\right)\right)+\nabla_{\theta_{di}}E_{\tau_{i}\in \Omega }\log Y_{di}\left(\tau_{i}\right)$.-**Optimize**: Compute losses $L_{\alpha },L_{g}$ and update coefficients α,β.
**3. Execute DQN algorithm**    Use the ϵ-greedy strategy to decide the action *a* as$$a=\left\{\begin{array}{cc} \arg\max Q(s,a), & P=1-\epsilon \\ \mathrm{random}, & \mathrm{other}. \end{array}\right.$$
-**If** $\sum_{n}\left[x_{n}\left(t\right)D_{n}\left(t\right)\right]\leq S$ **and** $\sum_{n}w_{n}f_{n}\left(t\right)\leq \psi$,
**then** calculate: [αC(t)+βη(t)]**else**, go back one step and re-decide action *a*.**end if**-**While** [αC(t)+βη(t)] is less than the revenue or the iteration count is greater than 200
    **do** output the maximum reward value max[αC(t)+βη(t)] and the optimal strategy $\arg\max_{\pi }\sum \mathrm{Pr}\left(\pi \to \pi^{\prime }\right)$

This paper defines trajectory preferences in the following three ways:

(1) The sampled knowledge bases of both the transmitter and the receiver are provided to human experts for comparison. The human experts return a binary feedback indicating whether $\tau_{m}≻\tau_{n}$. If the human experts cannot compare the results, this round is skipped, and the trajectory pair is not included in the KBPS.
(2) The adversarial neural network [42] returns a binary feedback based on existing human behavior to determine whether $\tau_{m}≻\tau_{n}$. Since the GAN is two-dimensional, a trajectory pair preference must be distinguished.
(3) Based on the generated reward function Q:S×A→Q (the reward function in this paper takes the form Q=αC(t)+βη(t), where generating the reward function refers to generating α and β), if the environmental feedback satisfies $\sum_{i=1}^{k}\gamma^{i-1}Q\left(s_{t+i}^{m},a_{t+i}^{m}\right)>\sum_{i=1}^{k}\gamma^{i-1}Q\left(s_{t+i}^{n},a_{t+i}^{n}\right)$, then $\tau_{m}≻\tau_{n}$.

The objective of GAN training is to develop a model that effectively simulates the logic of human preference [43], thus reducing the dependency on human experts. Furthermore, the GAN can correct erroneous human decisions caused by subjective factors. We train a discriminative neural network model $Y_{di}$, with its model parameters as $\theta_{di}$. This model learns the probability of selecting the best trajectory from the set of bias trajectories Ω. $\Omega_{np}$ represents the set of preferred trajectories and $\Omega_{all}$ represents all selected trajectories. The model parameter $\theta_{di}$ can be updated by using the stochastic gradient ascent algorithm: $\theta_{di}\leftarrow \theta_{di}+\nabla_{\theta_{di}}E_{\tau_{j}\in \Omega_{np}}\log\left(1-Y_{di}\left(\tau_{i}\right)\right)+\nabla_{\theta_{di}}E_{\tau_{j}\in \Omega_{np}}\log Y_{di}\left(\tau_{j}\right).$

The training process of the reward function is represented. We use human experts’ assistance and the GAN-assisted PbRL method to generate the reward function. First, human experts use the evaluation criteria to perform trajectory selection. Then, the selected bias data is used to train the adversarial neural network. If the adversarial network can correctly simulate the behavior of a human expert, it can be used as input for PbRL.

Then, we use the *Q*-function as the evaluation bias correctness indicator, where $Q\left(s,a_{t}\right)=\sum_{i}\gamma^{i-1}r\left(s_{t+i},a_{t+i}\right)$, assuming the bias preference, where *Q* increases as the function grows and is represented as

(12) $$\begin{array}{c} \hat{\rho }\left(\tau_{1}\to \tau_{2}\right)=\frac{1}{1+\exp[Q\left(s_{t}^{2},a_{t}^{2}\right)-Q\left(s_{t}^{1},a_{t}^{1}\right)]}. \end{array}$$

Here, $\hat{\rho }\left(\tau_{1}\to \tau_{2}\right)$ refers to the predicted preference to select the trajectory $\tau_{1}$ instead of $\tau_{2}$ after the intelligent agent computes the value *Q*. This trajectory is stored in the GAN-enhanced bias set of the form $\left(\tau_{1},\tau_{2},x=1\right)$. Then, we update the *Q*-value function and minimizes human bias in the *Q*-function based on the cross-entropy loss $L_{a}$, as shown below:

(13) $$\begin{array}{c} L_{a}=-\sum_{(\tau_{1},\tau_{2},\zeta )\in \Omega }\left[x\left(1\right)\log\hat{\rho }\left(\tau_{1}\to \tau_{2}\right)+x\left(2\right)\log\hat{\rho }\left(\tau_{2}\to \tau_{1}\right)\right]. \end{array}$$

In the adversarial neural network, $\rho \left(\tau_{1}\to \tau_{2}\right)=1-\rho \left(\tau_{2}\to \tau_{1}\right)$ generally holds, because the two endpoints are selected independently of the adversarial neural network, expressed as

(14) $$\begin{array}{c} \rho \left(\tau_{1}\to \tau_{2}\right)=Y_{di}\left(\tau_{1}\right), \end{array}$$

(15) $$\begin{array}{c} \rho \left(\tau_{2}\to \tau_{1}\right)=Y_{di}\left(\tau_{2}\right). \end{array}$$

Therefore, to better simulate human behavior, we also minimize the cross-entropy loss between the bias preference based on the *Q*-function and the GAN’s bias preference, which is represented by the auxiliary bias loss $L_{g}$:

(16) $$\begin{array}{cc} L_{g}=-\sum_{(\tau_{1},\tau_{2},x)\in \Omega }\; & \left[\rho \left(\tau_{1}≻\tau_{2}\right)\log\hat{\rho }\left(\tau_{1}≻\tau_{2}\right)+\left(1-\rho (\tau_{1}≻\tau_{2})\right)\log\hat{\rho }\left(\tau_{2}≻\tau_{1}\right)\right]\\ -\sum_{(\tau_{1},\tau_{2},x)\in \Omega }\; & \left[\rho \left(\tau_{2}≻\tau_{1}\right)\log\hat{\rho }\left(\tau_{2}≻\tau_{1}\right)+\left(1-\rho (\tau_{2}≻\tau_{1})\right)\log\hat{\rho }\left(\tau_{1}≻\tau_{2}\right)\right]. \end{array}$$

In this way, it can be predicted that the preference for selection based on the *Q*-function or the preference obtained through GAN will be higher. A higher preference score corresponds to a higher reward value.

Using GaPbRL, the reward function Q=αC(t)+βη(t) is obtained with the parameters (α,β), and the optimization problem becomes the dynamic storage optimization problem of the knowledge base. The action space includes the storage, deletion, replacement of knowledge blocks, and changes in the degree of extraction in the environment [44]. In this paper, actions are performed to sample the extraction process (ensuring that the extraction degree is an integer). We use an ϵ-greedy strategy to determine the actions, which can be written as

(17) $$\begin{array}{c} a=\left\{\begin{array}{cc} \arg\max Q(s,a), & P=1-\epsilon \\ \mathrm{random}, & \mathrm{other}. \end{array}\right. \end{array}$$

We use deep Q-networks to solve this optimization problem. First, the intelligent agent obtains the initial state distribution μ(s); next, the intelligent agent receives the state parameters $s_{t}=\left\{G_{t},W_{t}\right\}$ at time *t*, and the state parameters at the next time step. Due to storage capacity *S*, category volume *N*, and constraint of AoI, different strategies are required to modify the degree of knowledge extraction in $G_{t}$.

Under the assumption that the SKB deployment process is modeled as a finite constrained Markov decision process (CMDP), and the reward function Q=αC(t)+βη(t) is bounded, the proposed GaPbRL framework inherits the convergence properties of deep Q-networks (DQNs) under experience replay and target network stabilization. Specifically, the state space is finite with a limited number *N* of SKB categories; the action space is discrete, including cache, replace, and extract operations; and the reward is bounded where both C(t) and η(t) fall within the interval [0,1]. Therefore, the Q-function update in GaPbRL converges to a fixed point with probability 1 under standard stochastic approximation conditions.

The training stability of GaPbRL is ensured by two mechanisms: the target network and experience replay in the DQN, which reduce temporal correlation in updates; and the bounded adversarial loss in the GAN-based preference modeling, which prevents reward explosion. Since both C(t) and η(t) are bounded and normalized, the reward signal remains stable during training.

## 4. Simulation Experiments

Due to the knowledge graph-like characteristics of the SKB, most studies on semantic communication systems use knowledge graph-based knowledge bases. We use NELL-995, a large-scale knowledge base based on a knowledge graph, to evaluate the SKB deployment scheme proposed in this paper. NELL-995 consists of 754,920 entities and 200 types of relationships. In this paper, these entity–relation pairs are divided into 57 categories, corresponding to 57 types of knowledge base, with each category containing approximately 15 entity–relation pairs. The simulation assumes that all 867 entity–relation pairs can be retrieved from the cloud, and that the edge server can store at most 10 different categories of knowledge bases (setting N=10). The degree of extraction *M* of each knowledge base is a continuous value in the interval [0,1], which follows a Pareto distribution with parameter φ=1.3. The parameters are set as $k_{1}=0.75$, $k_{2}=0.25$, $k_{3}=-0.00054$, $k_{4}=0.67$, $k_{5}=-0.01$, $k_{6}=1.2$, and ε=0.05. The simulations are implemented based on the TensorFlow open-source platform and are conducted on a workstation equipped with an Intel(R) Core(TM) i5-12400F CPU @ 2.50 GHz, 16.0 GB RAM @ 3200 MHz, a 1 TB SSD, and an NVIDIA GeForce RTX 4080 SUPER GPU. In this paper, the definition of the average reward is given as follows:

(18) $$\begin{array}{c} \mathrm{average}\text{\_}\mathrm{reward}\left(X\right)=\frac{\sum_{x=1}^{X}\mathrm{reward}\left(x\right)}{X}. \end{array}$$

where *X* is the number of training rounds.

**Figure 3:**
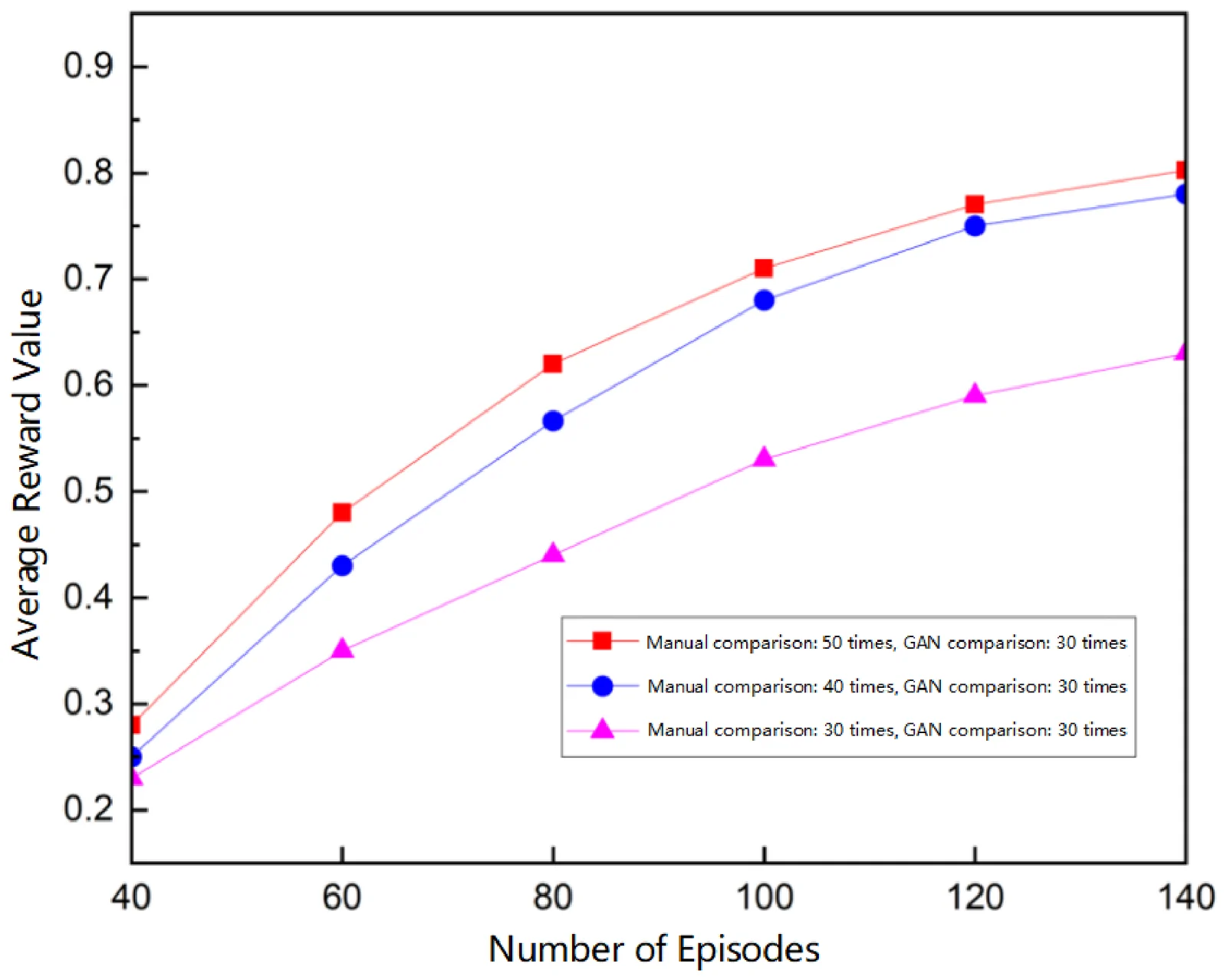
Comparison of average reward versus training episodes between GaPbRL-generated and conventionally designed reward functions.

Figure 3 illustrates a comparison of the average reward versus training episodes between GaPbRL-generated and conventionally designed reward functions. The average AoI threshold for each knowledge base is set to σ=15s, and the threshold for the weighted sum of the average AoI in all knowledge bases is set to Ψ=200s. The instantaneous AoI threshold for each knowledge base is set to λ=20s. The storage capacity of the edge server is set to S=120GB. The number of comparison trajectories is set to 50 for manual comparison and 50 for GAN-based comparison. The weighting coefficients of the output of the objective function of the PbRL reward function generation network are set to α=0.467 and β=0.533 (red curve). After multiple rounds of fine-tuning by human experts, the reward functions for the DQN algorithm are set to r=0.4C+0.6η (purple curve) and r=0.48C+0.52η (blue curve). It can be observed that when the GaPbRL-generated reward function is applied to the DQN algorithm, a clear convergence trend appears around the 60th and 80th training episodes, while the two reward functions manually designed by human experts exhibit clear convergence only around the 100th and 120th training episodes, respectively. It can also be observed that, in most training episodes, the average reward obtained using the GaPbRL-generated reward function is higher than the average weighted reward achieved by traditional manual-designed reward functions. From these observations, it can be concluded that the GaPbRL method proposed in this paper converges faster and yields a higher weighted sum of the knowledge-base hit rate and the degree of matching in the final output policy. The reason is that it is difficult for humans to design a reward function that is fully suitable for the DQN algorithm, and manual design typically relies on repeated fine-tuning and experimental trials. In contrast, the GaPbRL method can learn human preferences through the knowledge-base preference set ζ and the GAN-assisted preference set Ω, allowing the design of more accurate reward functions.

**Figure 4:**
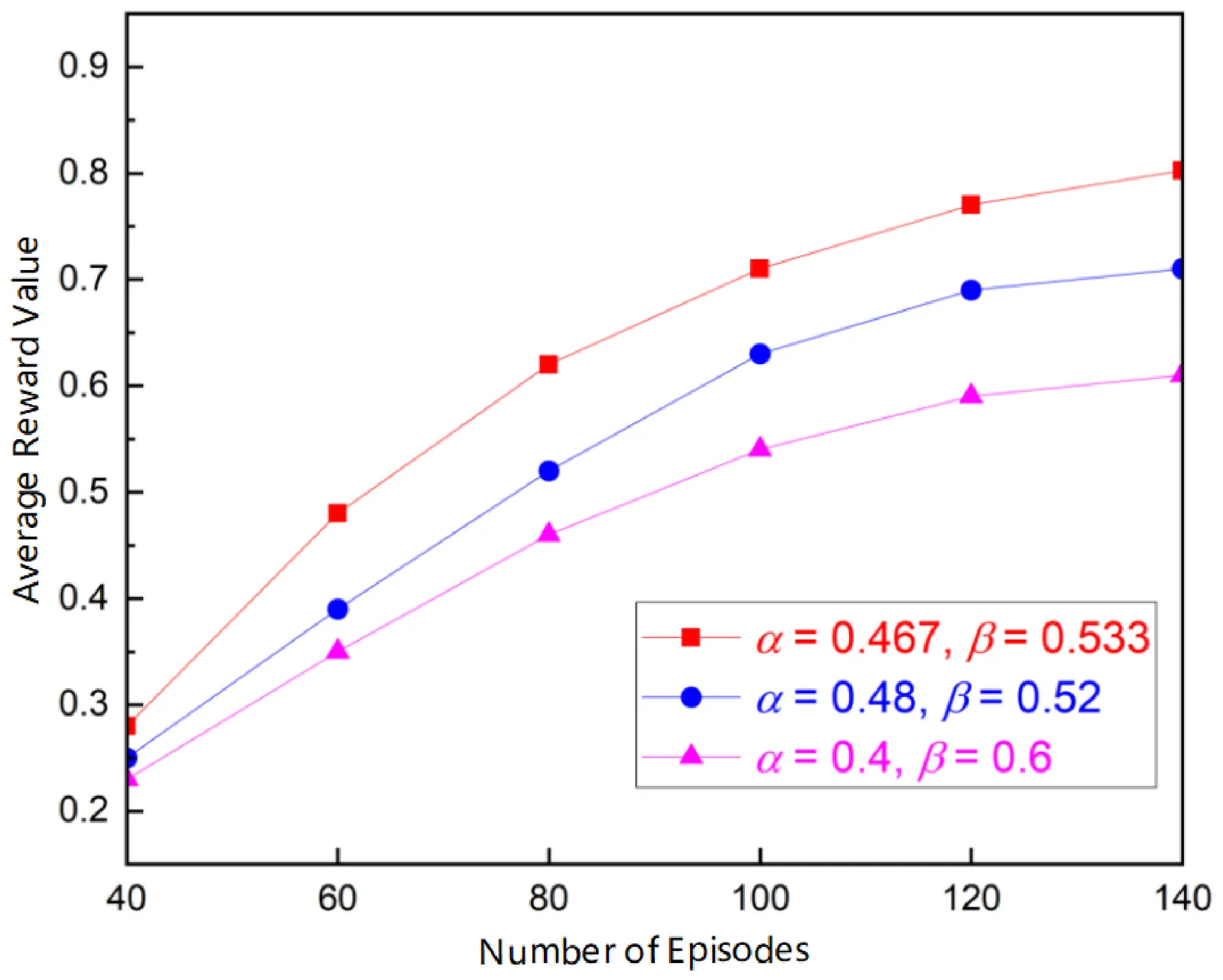
Relationship between knowledge-base hit rate and AoI under GaPbRL and conventional reward functions.

Figure 4 presents the relationship between the reward functions generated by GaPbRL and the conventionally designed reward functions in terms of the hit rate of the knowledge base versus instantaneous AoI. It can be observed that when the reward function generated by GaPbRL is applied to the DQN algorithm, the knowledge-base hit rate and AoI exhibit an exponential pattern that is consistent with the modeling assumptions in the problem formulation, without showing any obvious training bias. Moreover, the resulting knowledge-base hit rate η is generally higher than that obtained using traditional manual-designed reward functions. This is because, as time progresses knowledge bases with larger AoI may gradually become less aligned with user preferences, causing their rankings in the Pareto distribution to decline, which in turn reduces the knowledge-base hit rate.

**Figure 5:**
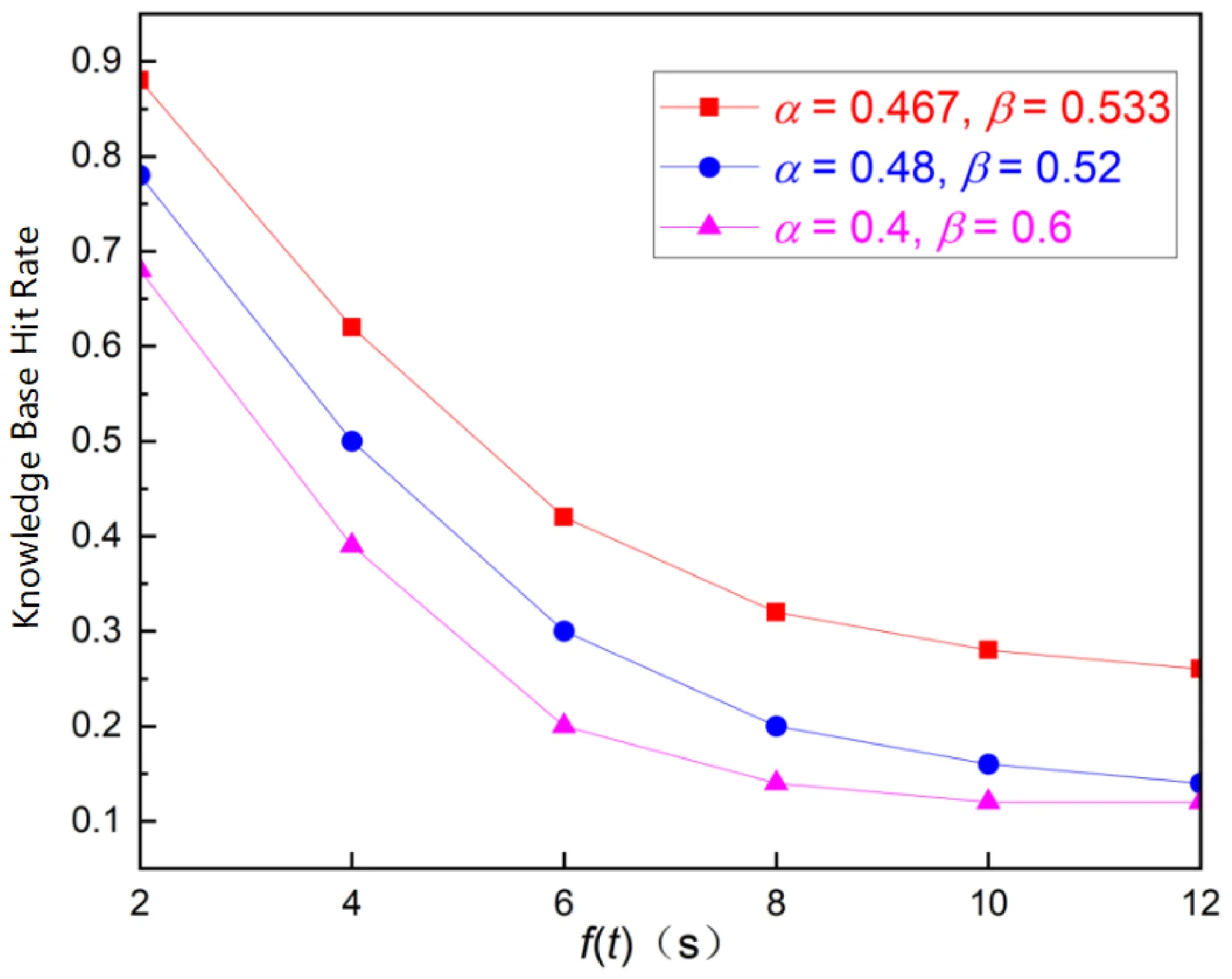
Average reward versus training episodes of GaPbRL under different numbers of human comparison queries.

Figure 5 illustrates the average reward versus training episodes of GaPbRL under different numbers of manual comparison trajectories. The numbers of manual comparison trajectories are set to 50, 40, and 30, respectively, while the number of GAN-based comparison trajectories is fixed at 30. When the number of manual comparison trajectories is 50 and the number of GAN-based comparison trajectories is 30, the weighting coefficients of the output of the objective function of the PbRL reward function generation network are α=0.462 and β=0.538 (red curve). When the number of manual comparison trajectories is 40 and the number of GAN-based comparison trajectories is 30, the weighting coefficients are α=0.441 and β=0.559. When the number of manual comparison trajectories is 30 and the number of GAN-based comparison trajectories is 30, the weighting coefficients are α=0.414 and β=0.586 (purple curve). It can be observed that as the number of manual comparisons increases, the resulting reward function becomes more accurate, which is reflected in faster convergence and higher precision. This indicates that the number of manual comparisons has a certain impact on PbRL, because a larger amount of preference data in the knowledge-base preference set ζ leads to better fine-tuning performance of the PbRL network.

**Figure 6:**
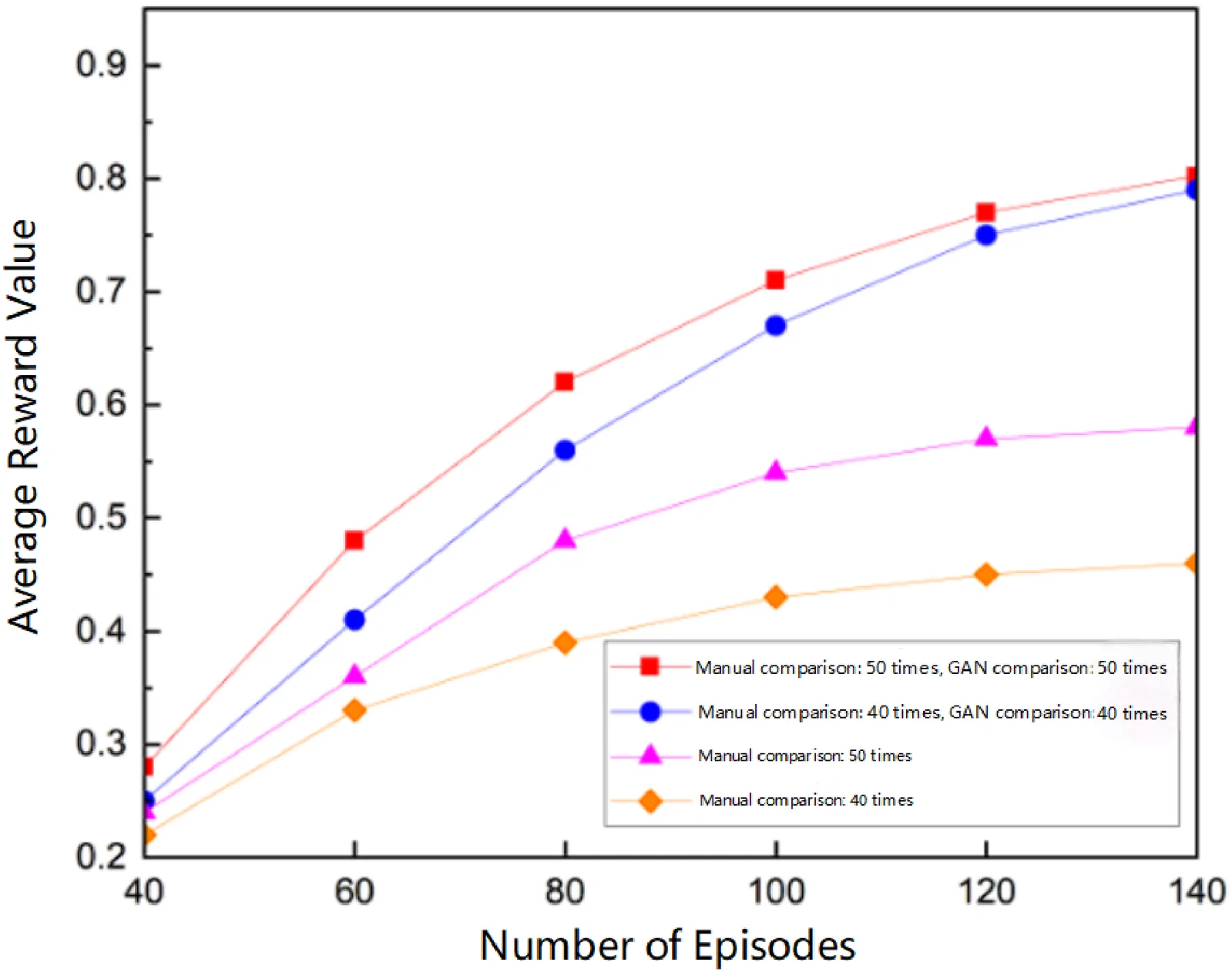
Average reward over episodes: Ablation of GAN-based preference generation in GaPbRL.

Figure 6 compares the average rewards versus the GaPbRL training episodes with and without the GAN-assisted preference set. Four groups of comparative experiments are conducted: (1) 50 manual comparison trajectories and 50 GAN-based comparison trajectories; (2) only 50 manual comparison trajectories; (3) 40 manual comparison trajectories and 40 GAN-based comparison trajectories; and (4) only 40 manual comparison trajectories. The weighting coefficients of the output of the objective function of the PbRL reward function generation network are set to α=0.467, β=0.533 (red curve); α=0.384, β=0.616 (purple curve); α=0.436, β=0.564; and α=0.681, β=0.319, respectively. The red and blue curves can be seen to exhibit convergence trends around the 60th training episode and achieve higher average reward values. Therefore, it can be concluded that, in PbRL, when the knowledge-base preference set ζ is fixed, the reward function generated with the assistance of the GAN-based preference set produces higher average rewards and converges faster during training. These results indicate that the GAN-assisted preference set plays a significant role in the PbRL network by helping to generate more accurate reward functions. The reason is that adversarial neural networks can learn human decision-making logic, further reducing the workload of manual comparisons in PbRL. By incorporating adversarial neural networks into the PbRL framework, manual costs can be reduced, leading to substantial economic benefits.

**Figure 7:**
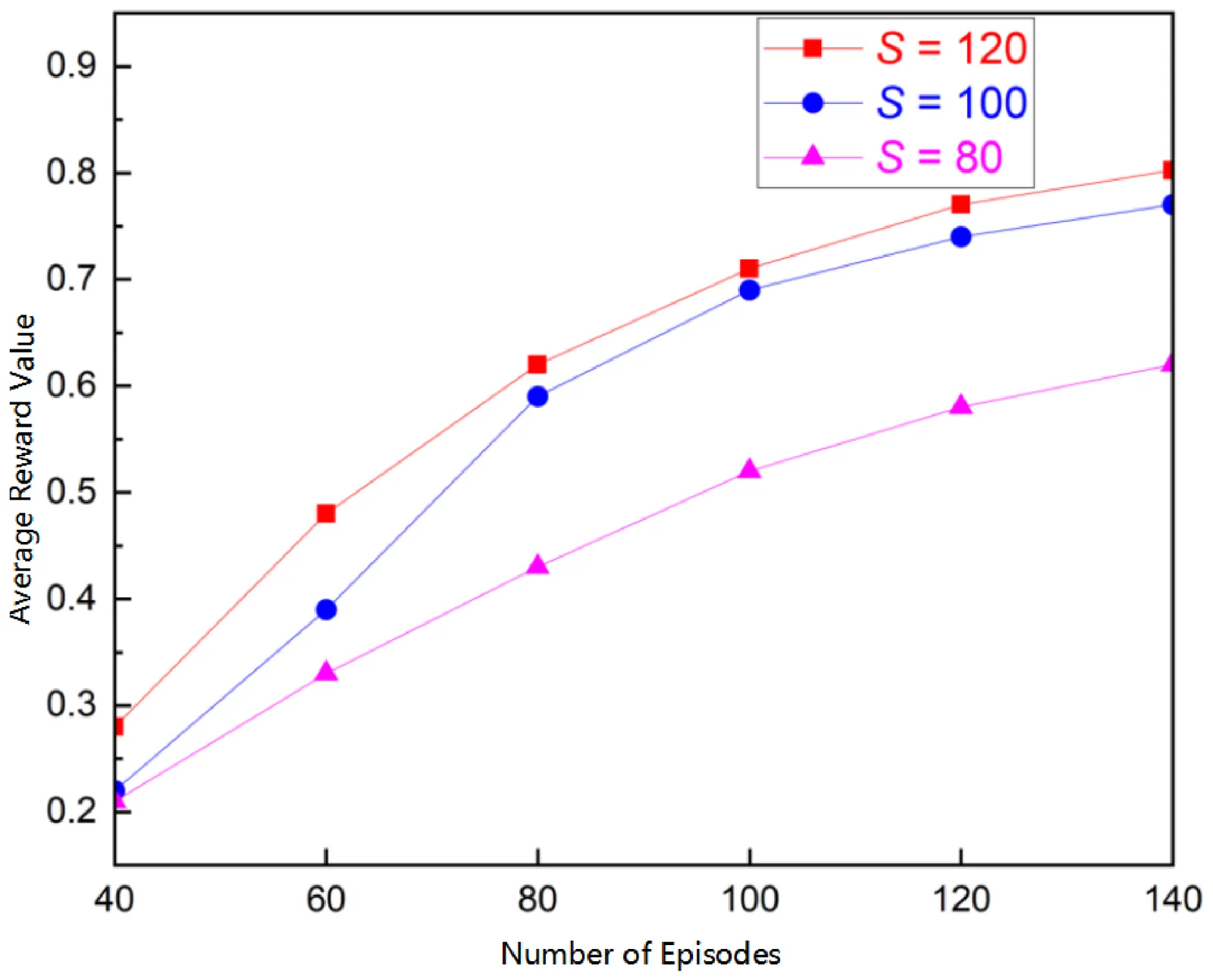
Impact of client storage threshold on average reward over episodes in GaPbRL.

Figure 7 compares the average reward versus the GaPbRL training episodes under different storage thresholds on the client. The storage capacity of the edge server is set to S=120, 100, and 80 GB. The numbers of manual comparison trajectories and GAN-based comparison trajectories are set to 50, and the weighting coefficients of the output of the objective function by the PbRL reward function generation network are α=0.467 and β=0.533. By observing the red and blue curves, we observe that the model exhibits clear convergence trends around the 60th and 80th training episodes, respectively. The final converged values of the red and blue curves are relatively close, indicating that when the client-side storage capacity is relatively sufficient, its impact on the average reward is limited. However, observation of the purple curve shows that when the storage capacity on the client is small, the convergence of the average reward is slower. Therefore, it can be concluded that the client storage capacity affects the convergence speed of the model. When the storage capacity in the client is sufficient, its impact on the convergence speed and accuracy of the model is relatively small. This is because sufficient client-side storage capacity provides a larger exploration space for the reinforcement learning agent to adopt greedy strategies. In contrast, when edge-server storage capacity is limited, although caching fewer knowledge bases may improve the degree of matching to some extent, it leads to slower convergence from a global perspective, and the objective function value of the optimal policy is also negatively affected.

**Figure 8:**
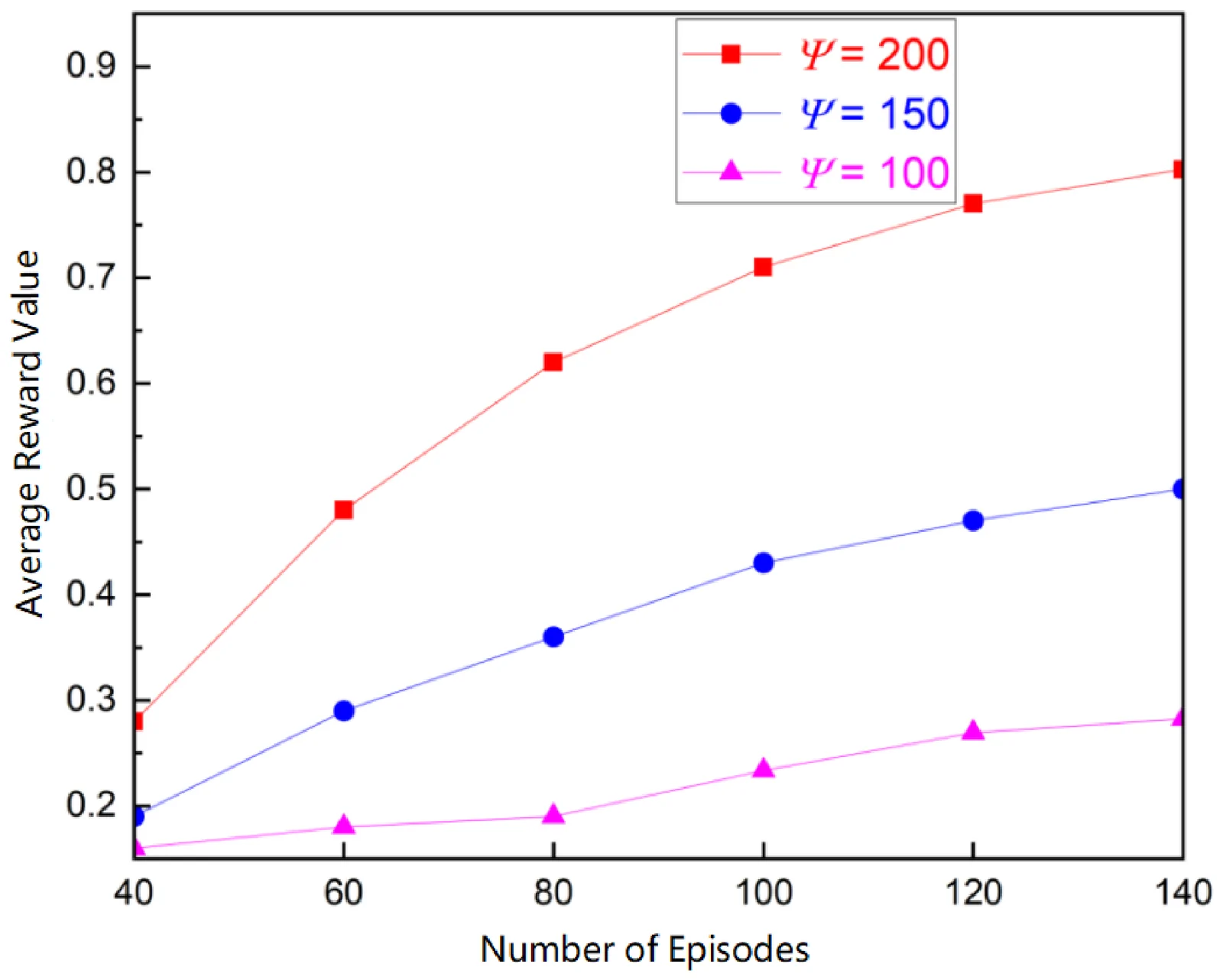
Effect of AoI weighted sum threshold on average reward over episodes in GaPbRL.

Figure 8 presents a comparison of the average reward versus the number of episodes for GaPbRL under different client-side storage threshold settings. The average AoI threshold for each knowledge base is set to σ=15s, the instantaneous AoI threshold is set to λ=20s, and the thresholds for the weighted sum of average AoI across all knowledge bases are Ψ=200,150, and 100s. The number of human comparison trajectories is set to 50, and the number of GAN-generated comparison trajectories is also set to 50. The weighting coefficients of the PbRL reward function generation network are α=0.467 and β=0.533. It can be observed that the red curve is higher than both the blue and purple curves, indicating that a larger threshold of the weighted sum of average AoI across all knowledge bases leads to a higher average reward. It can also be concluded that when this threshold is too low, the RL model exhibits weaker robustness. The reason is that a larger threshold expands the state space, allowing the agent to more easily explore suitable strategies. In contrast, when the threshold is too low, although the knowledge-base hit rate can be improved, the model can only converge to local optima, resulting in a lower global average reward.

**Figure 9:**
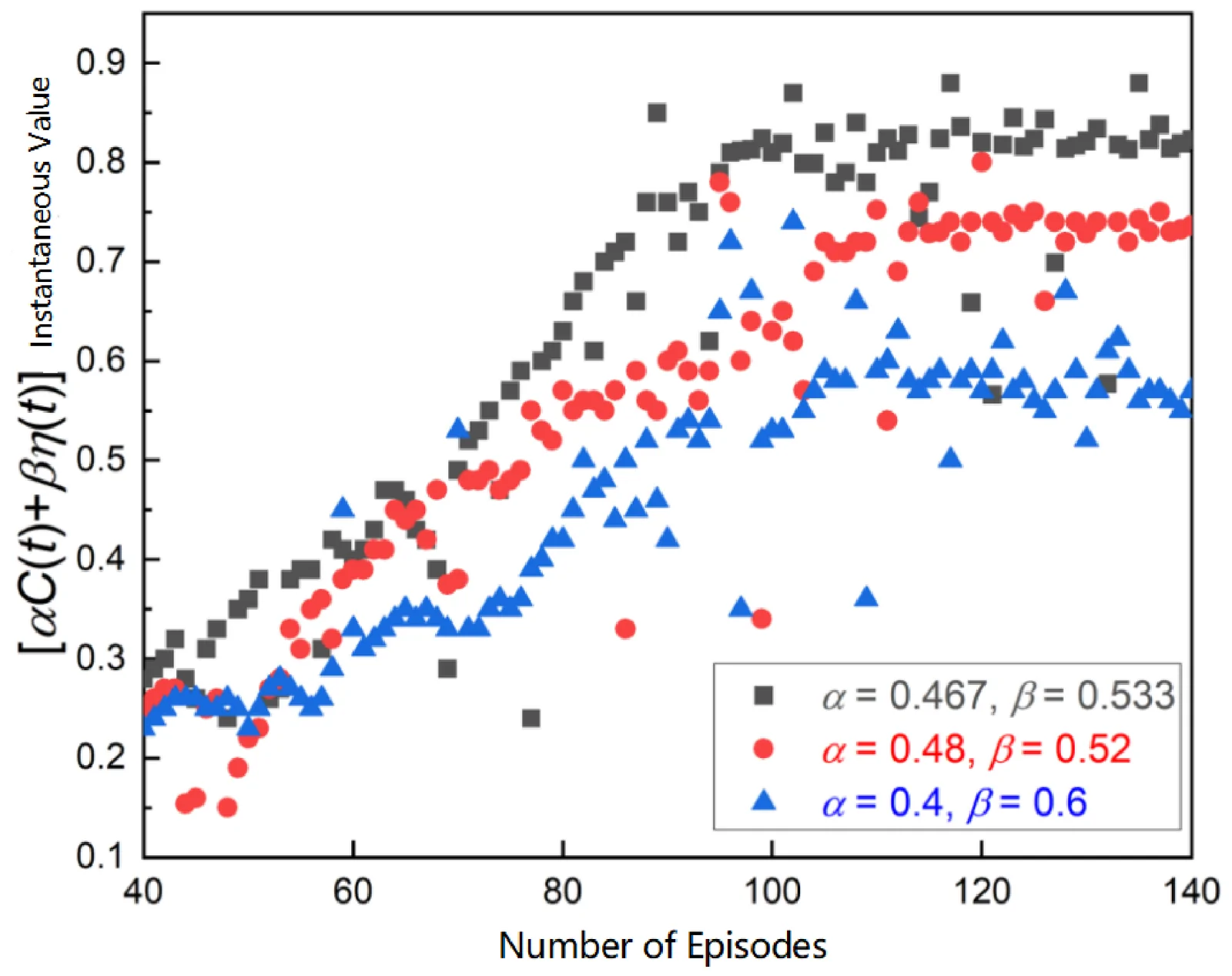
Comparison of instantaneous objective function values over training episodes for GaPbRL-generated and conventional reward functions.

Figure 9 illustrates the instantaneous value of the objective function versus the training episodes for the reward function generated by GaPbRL and the conventionally designed reward functions. It can be observed that, after parameter tuning, the trend of variation in the objective function value becomes relatively stable. When the GaPbRL reward function, corresponding to the square markers in the legend, with weighting coefficients of the output of the objective function of the PbRL reward function generation network set to α=0.467 and β=0.533, is applied to the DQN algorithm, the instantaneous value of the resulting objective function is generally higher than that obtained using manually designed reward functions (corresponding to circle and triangle markers) after approximately 40 training episodes. It can also be observed that the scheme proposed in this paper exhibits fewer abrupt drops in the instantaneous objective function value during training, indicating a higher training robustness.

**Figure 10:**
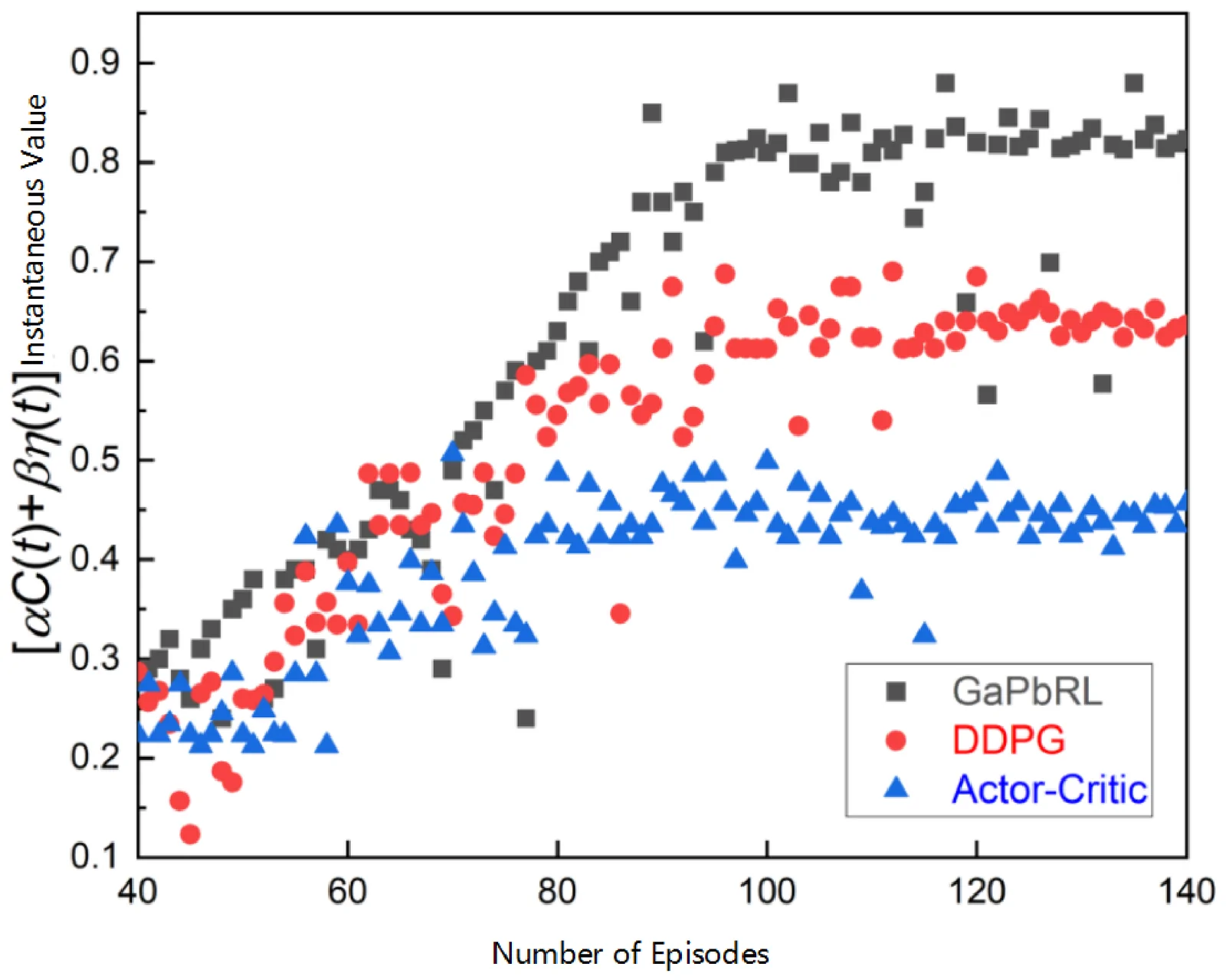
Instantaneous objective function over training episodes: GaPbRL versus baseline algorithms.

Figure 10 compares the instantaneous objective function value versus training episodes for GaPbRL and the DDPG and actor–critic algorithms. The average AoI threshold for each knowledge base is set to σ=15s, the threshold for the weighted sum of average AoI across all knowledge bases is set to Ψ=200s, and the instantaneous AoI threshold for each knowledge base is set to λ=20s. The storage capacity of the edge server is set to S=120GB. The weighting coefficients of the objective function output by the PbRL reward function generation network are set to α=0.467 and β=0.533. To make the comparison algorithms closer to the expert-tuned scenarios, the weighting coefficients of the comparison algorithms are set to α=0.4 and β=0.6. The learning rate of the actor network is set to $e^{-4}$ (approximately 0.018), the learning rate of the critic network is set to $e^{-3}$ (approximately 0.05), the discount factor is set to 0.99, and the batch size is set to 64. The soft update coefficient of DDPG is set to 0.001. It can be observed that the GaPbRL algorithm proposed in this paper converges to a higher weighted sum of the degree and hit rate of the knowledge-base matching. It can also be observed that GaPbRL exhibits fewer drops in the instantaneous objective function value during training, indicating higher robustness. Therefore, it can be concluded that the GaPbRL method proposed in this paper outperforms traditional reinforcement learning algorithms. This is because, compared with conventional RL methods, GaPbRL introduces a reward model training network and a generative adversarial network, enabling the agent to receive more accurate reward signals and thus better drive the retention, deletion, and replacement of knowledge-base categories. Based on all the experimental results above, it can be concluded that the GaPbRL method proposed in this paper can be effectively applied to the cloud–edge deployment problem of knowledge bases in semantic communication. Moreover, it demonstrates significant advantages in terms of knowledge-base hit rate, matching degree, and convergence speed of reinforcement learning models, while greatly reducing the workload of manual fine-tuning.

## 5. Conclusions

In this paper, we address the problem of dynamic knowledge-base deployment in semantic communication. First, the cloud–edge knowledge-base system model in semantic communication is introduced, including the internal structure of knowledge bases and the factors affecting the caching of the knowledge base. Next, the objective function and its constraints are formulated, followed by a proof that the optimization problem is NP-hard. Then, the GaPbRL method is presented to solve this optimization problem, including the Markov decision process for SKB replacement, the training of the generative adversarial network, the training of the PbRL reward function generation network, and the training of the deep Q-network. Finally, simulation experiments are conducted to verify the feasibility and superiority of the proposed approach. Future work will focus on practical validation in real-world dynamic networks and testbeds, as well as exploring low-overhead, scalable multi-agent edge collaborative solutions for ultra-large-scale SKBs.

## Data Availability

Data are contained within the article.

## Appendix A

The objective function is now divided into two sub-problems, based on the optimization variables and constraints, which are solved by linear decomposition methods (this decomposition is only for proving that the optimization problem is NP-hard and has no relation to the optimization method in Section 3):

(A1) $$\begin{array}{cc} \max\;\;\;\; & \lim_{T\to \infty }\frac{1}{T}\sum_{t=0}^{T-1}C\left(t\right)\\ s.t.\;\; & C1:\;\sum_{n}\left[x_{n}\left(t\right)D_{n}\left(t\right)\right]\leq S,\\ \; & C2:\;\sum_{n}x_{n}\left(t\right)\leq N,\\ \; & C3:\;x_{n}\left(t\right)\in \left\{0,1\right\},\;\forall n\in \left\{1,2,\ldots ,N\right\},\\ \; & C4:\;0\leq M_{n}^{r}\left(t\right)\leq M_{n}^{s}\left(t\right)\leq 1. \end{array}$$

(A2) $$\begin{array}{cc} \max\;\;\;\; & \lim_{T\to \infty }\frac{1}{T}\sum_{t=0}^{T-1}\eta \left(t\right)\\ s.t.\;\; & C1:\;f_{n}\left(t\right)\leq \sigma ,\\ \; & C2:\;\sum_{n}w_{n}\bar{f_{n}\left(t\right)}\leq \psi ,\\ \; & C3:\;\sum_{n}w_{n}=1,\;\forall w_{n}\geq 0,\\ \; & C4:\;0\leq f_{n}\left(t\right)\leq \lambda ,\;\forall f_{n}\left(t\right)\in Z. \end{array}$$

To prove that the optimization problem studied in this paper is NP-hard, the objective is to reduce the objective function or one of its subproblems to the *weighted K-set packing problem* (WKSP). The WKSP is a classical NP-hard problem. Given a universe $U_{e}$, where $\forall e\in U_{e}$, let *S* be a collection of subsets of $U_{e}$, and let C⊆S. The function Weight(s) denotes the weight of subset s∈S, and *k* represents the maximum allowed number of selected subsets [45]. The optimization problem can be formulated as follows:

(A3) $$\begin{array}{cc} \max\;\;\;\; & \sum_{s\in C}\mathrm{weight}\left(s\right)\cdot X_{s}\\ s.t.\;\; & C1:\;\sum_{s\in S}X_{s}\leq k,\\ \; & C2:\;X_{s}\in \left\{0,1\right\},\;\forall s\in S,\\ \; & C3:\;\sum_{e\in U_{e}}X_{e}\leq 1. \end{array}$$

Since constraint C4 in Equation (11) is an initialization constraint, the subproblem of the objective function [46,47] in this paper, defined in Equation (11), can be simplified as follows:

(A4) $$\begin{array}{cc} \max\;\;\;\; & \lim_{T\to \infty }\frac{1}{T}\sum_{t=0}^{T-1}C\left(t\right)\\ s.t.\;\; & C1:\;\sum_{n}\left[x_{n}\left(t\right)D_{n}\left(t\right)\right]\leq S,\\ \; & C2:\;\sum_{n}x_{n}\left(t\right)\leq N,\\ \; & C3:\;x_{n}\left(t\right)\in \left\{0,1\right\},\;\forall n\in \left\{1,2,\ldots ,N\right\}. \end{array}$$

By substituting the CMDP constraint equations into the subproblem optimization objective,

(A5) $$\begin{array}{cc} \max\;\;\;\; & \lim_{T\to \infty }\frac{1}{T}\sum_{t=0}^{T-1}\sum_{n}\left[k_{3}x_{n}\left(t\right)D_{n}\left(t\right)\right]\\ s.t.\;\; & C1:\;\sum_{n}\left[x_{n}\left(t\right)D_{n}\left(t\right)\right]\leq S,\\ \; & C2:\;\sum_{n}x_{n}\left(t\right)\leq N,\\ \; & C3:\;x_{n}\left(t\right)\in \left\{0,1\right\},\;\forall n\in \left\{1,2,\ldots ,N\right\}. \end{array}$$

It can be further simplified as follows:

(A6) $$\begin{array}{cc} \max\;\;\;\; & \sum_{t}\sum_{n}k_{3}x_{n}\left(t\right)D_{n}\left(t\right)\\ s.t.\;\; & C1:\;\sum_{n}\left[x_{n}\left(t\right)D_{n}\left(t\right)\right]\leq S,\\ \; & C2:\;\sum_{n}x_{n}\left(t\right)\leq N,\\ \; & C3:\;x_{n}\left(t\right)\in \left\{0,1\right\},\;\forall n\in \left\{1,2,\ldots ,N\right\}. \end{array}$$

To formally establish the NP-hardness of our subproblem defined in Equation (A6), we construct a polynomial-time reduction from the standard weighted K-set packing (WKSP) problem. We define the following mapping:

1. Each subset s∈S in WKSP corresponds to an SKB category *n* in our caching problem.
2. The weight of the subset weight(s) is mapped to the caching utility $k_{3}D_{n}\left(t\right)$ associated with retaining category *n*.
3. The cardinality constraint *k* (maximum allowed subsets) maps directly to the edge-server category limit *N*.
4. The binary decision variable $X_{s}$ maps to our caching control variable $x_{n}\left(t\right)\in \left\{0,1\right\}$.

Given this exact bijection, any polynomial-time algorithm capable of solving the SKB caching subproblem would simultaneously solve the WKSP problem. Since WKSP is known to be NP-complete, our proposed optimization problem is strictly NP-hard. This completes the formal proof.
